# *Streptococcus constellatus* Causing Empyema and Sepsis, Necessitating Early Surgical Decortication

**DOI:** 10.1155/2020/4630809

**Published:** 2020-07-12

**Authors:** D. Chrastek, S. Hickman, D. Sitaranjan, I. Vokshi, O. Kakisi, J. Kadlec, W. Bartosik, F. Van Tornout, V. Kouritas

**Affiliations:** Department of Thoracic Surgery, Norwich and Norfolk University Hospital NHS Foundation Trust, Norwich, UK

## Abstract

*Streptococcus constellatus* is an oropharyngeal commensal Gram-positive coccus, frequently associated with the respiratory tract. *S. constellatus* is part of the *Streptococcus anginosus* or *milleri* group, which has traditionally been considered to have propensity to cause empyema and purulent abscesses, a property that is sometimes overlooked as the severity of infections it causes may have a varying degree. In this case, we present the case of a 54-year-old male with known liver cirrhosis who developed a severe empyema during an acute liver failure episode, requiring extensive decortication and prolonged hospital admission.

## 1. Introduction

*Streptococcus constellatus* are small catalase-negative coccus, part of the normal flora of the mouth, gut, and urogenital tract but have been known to cause respiratory tract infections [1–3]. Although they are part of the commensal *Streptococcus milleri* group (SMG, also known as the *Streptococcus anginosus* group, SAG), its pathogenic potential is sometimes overlooked [4]. It has been known to cause parapneumonic effusions and abscesses but can normally be eradicated with intravenous antibiotics and occasionally minor surgical procedures such as chest drainage or washout [5]. We herein present the case of a 54-year-old male who developed acute liver failure and left chest empyema, necessitating early surgical intervention with extensive decortication for its treatment.

## 2. Case Presentation

A 54-year-old male was referred to our department for a left chest empyema following a week's history of progressively worsening shortness of breath, productive cough of white sputum, and an episode of frank hematuria. On presentation, he was visibly icteric with a tender and ascitic abdomen and reduced air entry on the left lung. His past medical history was significant for hepatitis C and liver cirrhosis, alcohol misuse, and a 35-year pack history of tobacco smoking. His family medical history was unremarkable.

**Figure 1:**
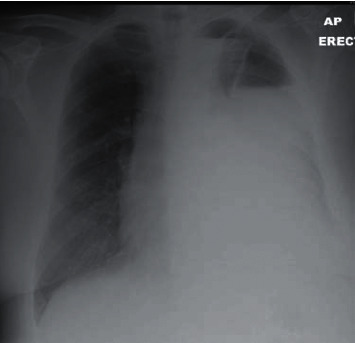
CXR on initial presentation.

**Figure 2:**
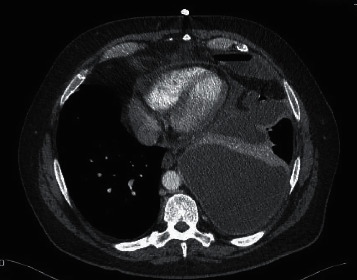
CT on initial presentation.

Initial CXR showed a large pleural effusion in the left lung (Figure 1). His chest-abdomen-pelvis CT revealed a large, multiloculated pleural effusion on the left side with almost complete collapse of the underlying lung (Figure 2). In his abdomen, ascites was confirmed, as well as hepatic changes consistent with chronic liver disease and an indeterminate hepatic lesion. Based on these findings, a 28 Fr chest drain was inserted initially, and the patient was started on IV piperacillin/tazobactam and clarithromycin on the respiratory ward. From the initial chest drain fluid sample, *S. constellatus* was grown (identified via Vitek MS, Biomerieux, France) and that was sensitive to all antibiotics tested, i.e., penicillin, clarithromycin, amoxicillin, clindamycin, tetracycline, and vancomycin (Vitek MS 2, Biomerieux, France, the disc diffusion method, interpreted according to EUCAST) [6]. Tissue samples were also sonicated prior to inoculation with 5% BA in order to increase the bacterial yield.

Upon admission, he was additionally found with acute liver failure (ALT 157 (U/L), Alk Phos 256 (U/L), Alb 18 (g/L), and INR 2.01). Three days after his admission and despite drainage and piperacillin/tazobactam, he clinically deteriorated becoming septic (according to the Society of Critical Care Medicine criteria—sepsis as having two or more criteria of systemic inflammatory response and with an infection source confirmed or suspected) [7]. For this, he was urgently referred to our department for review during which a surgical intervention was decided. Before surgery, his coagulation imbalances (INR 1.94) were corrected to an extent, with vitamin K initially (10 mg IV 3 doses) and then with fresh frozen plasma (4 units perioperatively).

**Figure 3:**
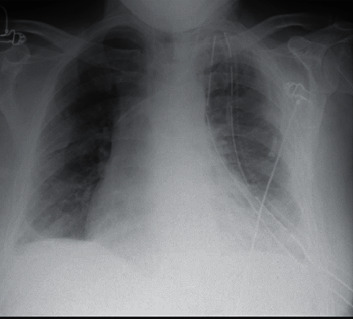
Immediate postoperative CXR.

He underwent VATS exploration with a 3 cm lateral utility incision over the dependent area and 1 camera port anteroinferiorly. Upon entrance into the chest, 1 litre of foul smelling pus was evacuated. A thick mature cortex was noted on the lung, prohibiting it from expansion. A clear plane between the lung and the cortex was created, and therefore, a decortication was performed. Two wide chest drains (28 Fr anterior and tunnelled 32 Fr posterior) were left into the chest, and the lung reexpanded totally after the procedure with minimal air leak (Figure 3). The samples taken during the procedure also grew *S. constellatus*. His postoperative recovery from his chest point of view was uneventful with the anterior drain removed 3 days later. On admission, he was screened for blood-borne viruses and was also found positive for hepatitis C virus (HCV), for which a viral load showed active disease, and he was referred for treatment. He was eventually discharged home without drains, 2 weeks after his operation, remaining in-hospital mainly for investigation of his hepatic lesion.

**Figure 4:**
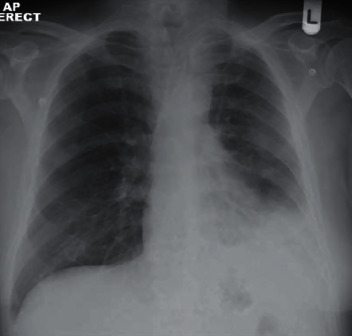
CXR on readmission.

**Figure 5:**
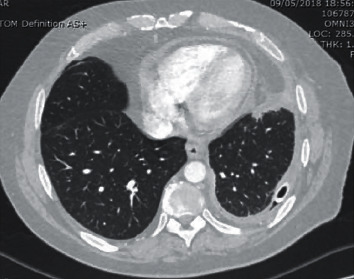
CT after readmission and redrainage.

**Figure 6:**
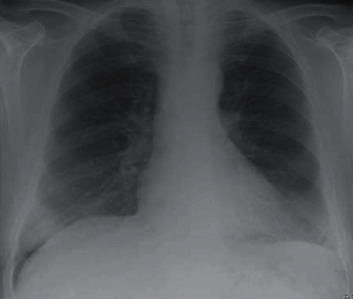
Repeat CXR from last outpatient assessment.

Two months later, he was readmitted due to raised inflammatory markers and pus leaking from the drain site. Based on the CXR (Figure 4), a surgical 28 Fr drain was reinserted, and he was again started on antibiotics. A CT scan was repeated showing ongoing left-sided consolidation (Figure 5) with small pleural collection. The pus swab taken at this time showed negative for growth of any bacteria, yeast, or fungi. Conservative treatment was decided, and he was finally discharged home with oral antibiotics a couple of days later. Six months after his procedure, he remains well from his chest point of view without any further relapses of his chest infection or pleural collection (Figure 6).

## 3. Discussion

We herein present a case in which *Streptococcus constellatus* caused a difficulty in treating severe empyema in an immunocompromised patient. The pathogenic potential of commensal bacteria such as *S. constellatus* should not be underestimated especially in patients with immunosuppression, making an aggressive, possibly surgical, treatment necessary. The subspecies *S. constellatus* is known to be a causative organism of respiratory infections, occasionally complicated by easily treatable pleural effusions or abscesses.

A study by Clarridge et al. [5] found that of 41 patients with abscesses caused by *S. constellatus*, the majority (26/41) had infections of the soft tissues, while only 7 cases had pleuropulmonary disease. To add to this, Noguchi et al. [8] studied a cohort of 30 patients with SMG infection, while Kobo et al. [9] studied a cohort of 236; both studies concluded that the *S. constellatus* subspecies was less associated with empyema and less severe infection than other SMG subspecies, such as *S. intermedius*. The patient in our case report, however, suffered from an aggressive *S. constellatus* infection, likely due to immunosuppression secondary to severe hepatic disease caused by hepatitis C, alcoholic cirrhosis, and what was later found to be hepatocellular carcinoma. Literature suggests that while *S. constellatus* is a known causative organism in respiratory and pleural infections, its pathogenicity is relatively less severe in comparison to its other SMG siblings. Kobo et al. [9] found interestingly that *S. constellatus* was associated more with bacteremia than with abscess or empyema formation. Clarridge et al. [5] reported that *S. constellatus* infections were sufficiently treated with simple drainage and intravenous antibiotics. Junckerstorff et al. [10] reported that patients growing *S. constellatus* had a shorter hospital stay and a lower 30-day mortality rate than other SMG organisms. There is one other case report by Che Rahim et al. [11] that describes a case of empyema caused by *S. constellatus*, for which decortication was required following a 6-week course of IV antibiotics, with improvement afterwards and no recurrence or sequelae.

In contrast, a surgical intervention was necessitated in our case early on the onset of events because of clinical deterioration in a short period of time because of the liver failure. Also, in the case of our patient, even after treatment with IV antibiotics and an extensive decortication operation, he was readmitted for further issues with his chest. We believe that the likely cause of the severe clinical manifestation of a usually mild organism was due to the patient's other comorbidities, namely, decompensated liver failure and later discovered, hepatocellular carcinoma, as contributing to an overall state of immunosuppression.

Initially, the plan had been to postpone the surgery until the sepsis had settled, and he was haemodynamically stable. Unfortunately, due to his deterioration, he was taken to theatre, and since the cortex was easily removable, a decision was made intraoperatively to complete an extensive decortication in favour of a simple washout and a possible return to theatre. A VATS procedure was more appropriate than open, especially due to the existing coagulation abnormalities and to the overall deteriorated condition of the patient.

In conclusion, *Streptococcus constellatus* normally has relatively low pathogenic potential; however, in this case of an immunocompromised patient, it resulted in sepsis and required extensive decortication. Sepsis is defined as infective source confirmed or expected with 2 or more of the following criteria: temperature >38°C or <36°C, heart rate >90 beats per minute, respiratory rate >20 respirations per minute or PaCO_2_ <32 mmHg, and WBC >12,000/mm³, <4,000/mm³, or >10% bands (7). Regardless of the organism grown, it is always important to consider the potential for simultaneous comorbidities to create an immunocompromised state in patients, which may make early surgical intervention necessary.

## Abbreviations

IV: Intravenous
CXR: Chest X-ray
Fr: French
CT: Computed tomography
ALT: Alanine transferase
Alk Phos: Alkaline phosphatase
Alb: Albumin
INR: International normalized ratio
VATS: Video-assisted thoracic surgery.
